# A Blessing or a Curse

**Published:** 1875-10

**Authors:** 


### A BLESSING, OR A CURSE?

An English physician, in a lecture to a female audience on the use of alcoholic beverages, asserted that the "babes of London are never sober from their birth until they are weaned."

The use of beer and ale among nursing mothers is perhaps not so common in the United States as it is in England, but it is by far too common. How often a friend, and even the family physician, will recommend the use of beer to the mother, not only to give tone to the system, but as the means of nourishing the child.

What a fatal mistake! The eternities, with their mysteries, alone can reveal the amount of damage resulting from so dangerous a practice. The stimulant thus taken by the mother readily enters into the food nature has provided for the child, and every particle of nourishment drawn from the life-giving fountain is impregnated with a substance that is not only foreign to the highest physical condition of the child, but is actually poisonous to the system.

The old theory that these drinks are necessary to the well-being of the mother and the sustenance of the child, is thoroughly exploded, and those who advocate these notions are far in the rear of the car of progress. It is a well established fact, demonstrated by the most logical minds of the day, that the physical system is in the most healthful and natural state when freest from the influence of stimulants.

Besides the custom being entirely unnecessary and uncalled for, every mother should take into consideration the future welfare of her child. There can be no doubt but that the appetite for stimulants is often bred and nurtured at the mother's breast. Regarding this as true, how can any mother for a moment listen to the advice of physician or friend in a matter of such weighty import to the child.

Mothers! in taking that draught that seems so harmless to you, remember that you are doubtless paving the way to a drunkard's doom for your darling child.

Remember, that the beer or wine or whiskey which you drink, tinctures at once the milk in your breast, and produces the same effect upon the brain of your babe, which you feel when you have swallowed the potent poison.

Remember, that these beverages are not food, do not nourish, give no real strength; but do possess great power to weaken and destroy.

Remember, that you have the power to make your child great or small, a blessing or a curse to the world.

---

SALT is a simple remedy for many things. It will cure sick headache, make cream freeze, make the butter come, take ink-stains out of cloth of any kind, kill wens, kill worms, make the ground cool, so it is more congenial to celery, cabbage, &c. It will ease the itching pain caused by irritable skin-diseases, like hives, itch, &c. It will produce vomiting or stop it, as you like; and many other things too numerous to mention. All pure salt will do this to a certain degree, but sea salt is the most effectual in its action.

